# Chemical Recycling of End-of-Life Tires Using Catalytic Pyrolysis: Effects of Catalysts and Process Conditions toward the Production of a Highly Aromatic Pyrolysis Oil

**DOI:** 10.1021/acs.iecr.5c02163

**Published:** 2025-09-26

**Authors:** Stylianos D. Stefanidis, Eleni Pachatouridou, Eleni Heracleous, Angelos A. Lappas, Iacovos A. Vasalos

**Affiliations:** 1 Chemical Process & Energy Resources Institute (CPERI), Centre for Research and Technology Hellas (CERTH), 6th km Harilaou-Thermi, Thessaloniki 57001, Greece; 2 School of Science and Technology, International Hellenic University, 14th km Thessaloniki-Nea Moudania, Thessaloniki 57001, Greece

## Abstract

End-of-life tires (ELTs) represent both an environmental challenge and an opportunity as an untapped resource for materials and energy recovery. This work demonstrates the catalytic pyrolysis of ELTs using equilibrium FCC catalysts with varying metal contamination levels and ZSM-5 catalyst additives to produce highly aromatic oils with considerable promise for direct application. The investigation employed a systematic approach, screening catalysts in a batch reactor followed by validation in a continuous process development unit. The produced oils were characterized by quantitative methods to determine aromatic hydrocarbon yields and catalyst selectivity. Results showed strong agreement between the two setups with equilibrium FCC catalysts achieving 48 wt % oil yields containing up to 87 wt % aromatic hydrocarbons. Ni contamination on the catalyst shifted selectivity from monoaromatic to polyaromatic hydrocarbons and increased the overall aromaticity, rendering the oil attractive as a feedstock to produce carbon black and as a source of BTX and aromatic fuel additives.

## Graphical abstract

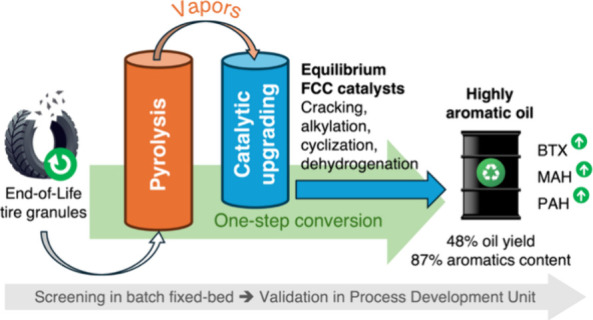

## 1 Introduction

Mobility is a vital aspect of modern life and the global economy, and tires play a crucial role in enabling it. It is estimated that by 2030, 1.5 billion tires will be produced worldwide each year, of which approximately 1.2 billion tires will reach the end of their useful life annually^1^ and will be classified as end-of-life tires (ELTs). Managing ELTs presents a significant environmental and economic challenge. Tires are composite products composed of natural and synthetic rubber (40–65 wt %), carbon black (21–22 wt %), reinforcing steel wires, textile fibers, vulcanization agents, oils, and other additives.^2–4^ Traditional disposal methods, such as landfilling and incineration, are unsustainable due to land scarcity and the emission of greenhouse gases and toxic pollutants. Conventional recycling approaches for ELTs are not circular, as the materials recovered are mainly used in other industries rather than being reincorporated into new tires.^5^ Within the circular economy framework, pyrolysis has emerged as the missing link between ELT management and the tire industry, offering a pathway to produce secondary raw materials that can be reused in tire manufacturing and other high-value applications.^6^

Thermochemical conversion technologies, such as pyrolysis, are emerging as promising methods for valorizing ELTs. Pyrolysis involves heating under an inert atmosphere, causing the rubber polymers and other organic components to break down into vapors composed of reactive radicals. These vapors undergo complex reactions including chain scission, hydrogen abstraction, and aromatization. Rapid quenching of the vapors produces a liquid known as thermal pyrolysis oil (TPO), which is a complex mixture of hydrocarbons.^4^ The optimum pyrolysis temperature frequently reported is around 500 °C, with TPO yields in the range of 45–65 wt %,^7^ strongly depending on the feedstock composition and other reaction parameters, such as pressure, heating rate, carrier gas, and residence time, which in turn can be influenced by the reactor technology.^1^ Several reactors types have been reported in the literature for the pyrolysis of ELTs.^7^ Fixed-bed reactors are characterized by slow heating rates and are challenging to scale up but are simpler in construction and operation and are commonly employed in laboratory settings. Fluidized-bed reactors on the other hand, pioneered for polymer pyrolysis by Kaminsky in Hamburg,^8^ offer higher heating rates, low residence times, and uniform reactor temperatures. Recent advances demonstrated the scalability of ELT pyrolysis from pilot scale (TRL-5) to semi-industrial plants (TRL-7), with auger reactor technology showing particular promise for continuous operation and consistent product quality.^9^

The compositional complexity of TPO, with high levels of sulfur, nitrogen, polyaromatic hydrocarbons (PAHs), and heavy-molecular-weight structures with boiling points exceeding 350 °C, limits its direct application as a fuel.^10–12^ Recently, due its similarities with petroleum streams, coprocessing TPO in existing refinery units has attracted significant attention as a promising pathway for its utilization.^6^ Nonetheless, the variance in quality that is inherent in waste-derived products and difficulty determining its composition with conventional characterization methods can present barriers for its adoption without prior separation and purification that increase in complexity with increasing TPO heterogeneity. Recent work in a pilot-scale plant showed that distillation of TPO achieved BTEX concentrations up to 85.5 wt % in light fractions, while heavy fractions with high carbon-to-hydrogen (C/H) ratios and PAH content were obtained.^13^ Such heavy oils are promising as alternative feedstocks in the furnace process that produces carbon black used in tire manufacturing^13,14^ and conventionally derived from petroleum-based oils or coal tar, where high aromaticity is preferred to produce high-quality carbon black grades.^15^ In this regard, the aromaticity and presence of PAHs in TPO are advantages, as they facilitate the nucleation and growth of carbon black particles.^14^

As an alternative to pyrolysis, catalytic pyrolysis with zeolites offers enhanced selectivity by enabling targeted production and increased yields of specific product types. By tailoring catalyst properties such as acidity and pore structure, the process can direct the formation of desirable compounds, resulting in catalytic pyrolysis oils (CPOs) with improved composition and properties relative to TPO.^16^ In this process, vapors generated from the thermal breakdown of ELTs are exposed to a solid catalyst, promoting the cracking of heavy molecules and the selective formation of desirable products. Appropriate catalyst selection and design can enable the selective production of valuable molecules like naphtha and middle-distillate-range hydrocarbons. Additionally, catalysts can facilitate the removal of heteroatoms, further improving the quality and applicability of CPO. ELT catalytic pyrolysis has been studied primarily using zeolites, which are microporous aluminosilicate materials with a large internal pore surface area that offers a high concentration of active sites for the conversion of reactants.^17^ The most frequently studied are the Y and ZSM-5 types, although MOR, beta, and other types have been explored as well. Catalytic pyrolysis with zeolites generally reduces the CPO yield in favor of gas products and coke formation.^18–23^ However, zeolites have been consistently found to yield less complex and more aromatic oils compared with thermal pyrolysis. Comparative studies of Y and ZSM-5 zeolites for ELT pyrolysis are generally in agreement that Y zeolite exhibits higher cracking activity, causing a more pronounced reduction of the CPO yield compared to ZSM-5,^19–21^ and more coke formation.^18,19,24^ Nonetheless, Y zeolites reportedly result in higher BTX^19–21^ and PAH yields,^19,25,26^ producing overall more aromatic CPOs.^18,25–27^ Due to the higher cracking activity, CPOs from Y zeolites contain a higher portion of compounds in the naphtha and middle-distillate range.^19,21,28^ ZSM-5, on the other hand, exhibits lower cracking activity and produces less coke^18,19^ but is more selective toward gas products,^19,21,25,26,29^ BTX,^25,26,30^ and monoaromatic hydrocarbons (MAHs).^29^ Other zeolite types were also effective for the cracking of ELT vapors and the formation of aromatics, with their activity and selectivity primarily dictated by their pore network and the type and strength of acid sites.^31^ Zeolites promoted with metals have been studied as well; several groups reported enhanced cracking activity and increased formation of BTX, MAH, and naphthalene with zeolites modified with Fe,^28,32–34^ Zr,^28^ Cu,^33^ Co,^33^ Zn,^33,35^ Ni,^33,34^ and Ga,^36^ as well as sulfur reduction with Cu and Pt impregnated zeolites.^37,38^ Reduced selectivity to PAHs was reported with Zr-, Fe-,^28,34^ and Ni-modified zeolites.^34^

Equilibrium FCC catalysts have recently garnered attention as potential ELT catalytic pyrolysis catalysts, with overall reported effects similar to those of zeolite catalysts.^29,39–42^ However, these studies were either limited to kinetic investigations^41^ or carried out in stirred tank and closed rotary kiln reactors not optimal for maximizing liquid hydrocarbon yields.^29,39,40,42^ Equilibrium FCC catalysts are used catalysts withdrawn from refinery FCC units to make room for fresh catalyst to be added. They are composite materials containing Y zeolite as the main active phase and remain active^43^ but may be partially deactivated and contaminated with metals like Ni and V from petroleum feeds. Using equilibrium FCC catalysts in ELT pyrolysis offers a cost-effective alternative to fresh catalysts and helps address the disposal issue of large quantities of these catalysts that are generated globally.^44–46^ While metal contamination is detrimental in FCC processes because it promotes dehydrogenation and shifts the selectivity toward more aromatic products and coke, it may be beneficial in ELT pyrolysis to boost the yield of aromatics.

Although numerous studies have investigated the catalytic pyrolysis of ELTs, the majority were of a preliminary nature, predominantly utilizing micropyrolyzers, laboratory-scale fixed-bed batch reactors, or batch tank reactors. As recently pointed out,^16^ studies adopting more advanced continuous systems remain scarce even though such approaches better approximate industrially relevant conditions and provide perspectives for practical implementation. Studies on the catalytic pyrolysis of ELTs using equilibrium FCC catalysts are also few in number, while the catalytic effect of metal poisoning typically encountered in these refinery waste streams has not been previously explored. This study addresses both gaps in the literature by investigating the catalytic pyrolysis of ELTs in both batch and continuous systems using equilibrium FCC catalysts featuring varying levels of metal poisoning and ZSM-5 catalyst additives. The catalysts were screened in a batch system featuring a fixed-bed reactor, and the most promising catalysts were then validated in a continuous process development unit (PDU) featuring a two-stage fluidized bed reactor system employing variable catalyst-to-feed (C/F) ratios. Effort was made to maintain similar vapor–catalyst contact times in both setups, enabling a direct comparison that revealed insights into the impacts of scale and associated process parameters on product yields. The resulting pyrolysis oils were thoroughly characterized via quantitative analysis methods, revealing aromatic hydrocarbon contents up to 87 wt %, the highest reported to date from studies employing continuous systems, remarkably achieved using low-cost equilibrium catalysts. This exceptional aromaticity holds considerable promise for direct practical application; the heavy oil fraction can serve as a feedstock to produce carbon black without the need for further upgrading to increase its aromaticity, while the light fraction presents a compelling source of BTX hydrocarbons and aromatic SAF additives.

## 2 Experimental Section

### 2.1 Feedstock

The feedstock (ELT) was provided by Michelin and was described as granules of multibrand all-tire with a particle size between 0.2 and 0.8 mm. The feedstock was free of textile and steel reinforcement materials.

### 2.2 Catalysts

The catalysts used in this work were two equilibrium FCC Y zeolite-based catalysts (USY and Ni/USY) and two ZSM-5 zeolite-based catalysts (ZSM-5 and Co/ZSM-5). All catalysts originated from commercial refinery catalysts and were composite materials composed of a microporous zeolite phase embedded in a mesoporous matrix. USY and Ni/USY were both equilibrium FCC catalysts; the former had negligible Ni poisoning, while the latter exhibited significant Ni poisoning. ZSM-5 was derived from an unused commercial ZSM-5 catalyst additive after being steamed at 796 °C for 9 h. Steaming aimed to simulate typical hydrothermal deactivation that takes place during catalyst use and lower the acidity of the unused ZSM-5 catalyst additive to those of the used equilibrium FCC catalysts. Co/ZSM-5 was derived from wet impregnation of an unused ZSM-5 catalyst additive with aqueous solutions of Co­(NO_3_)_2_·6H_2_O to achieve 5 wt % Co loading.^47^

### 2.3 Experimental Setups and Procedures

Catalyst performance was evaluated in two distinct experimental setups: a batch bench-scale fixed-bed reactor and a continuous process development unit (PDU). Both systems featured separate sections for the pyrolysis of ELT and catalytic upgrading of the resulting vapors, with no contact taking place between the ELT and the catalyst. The bench-scale system was utilized for the initial rapid screening of the catalysts at a fixed C/F ratio. The most promising catalysts were then evaluated in the PDU employing variable C/F ratios with continuous feeding of the ELT feedstock and catalytic upgrading of the vapors at variable C/F ratios.

The bench-scale system (Figure 1) consisted of a stainless-steel fixed-bed reactor, a head housing a piston for the introduction of the feedstock in the reactor, a three-zone electrical furnace for the heating of the reactor, a glass trap submerged in a cooling bath maintained at −10 °C for the collection of the liquid products, and a system for the collection and volumetric measurement of the gas products via liquid displacement.

**Figure 1:**
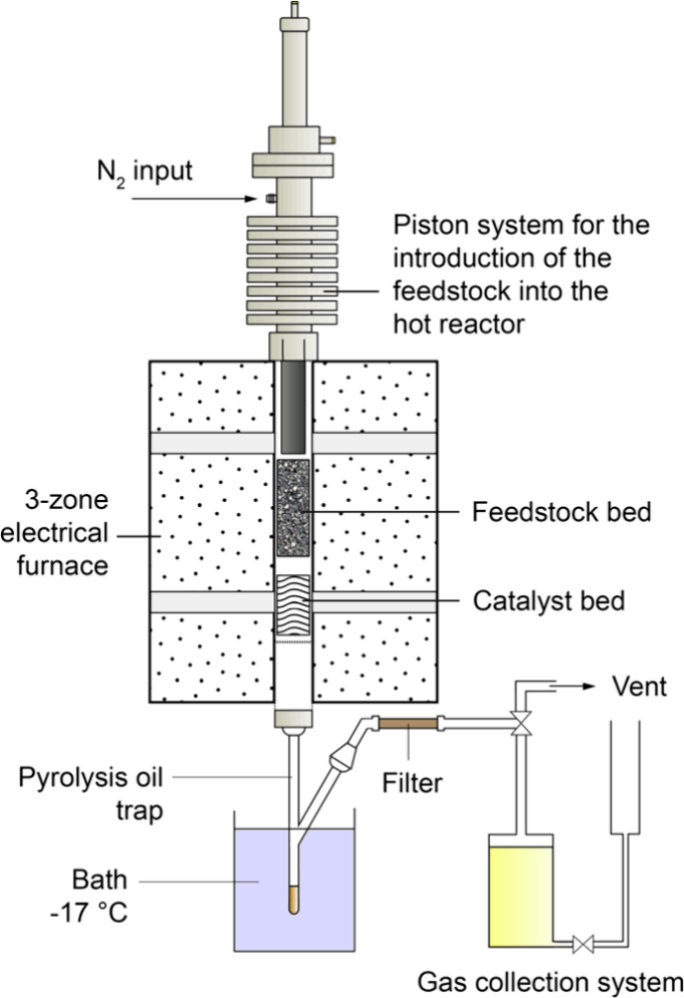
Schematic representation of the batch bench-scale fixed-bed experimental setup.

For each test, 2.8 g of ELT was loaded in the reactor head, while 8.4 g of catalyst (C/F = 3) was loaded in the reactor, both between two pairs of quartz wool layers for support. The head was secured atop the reactor, which was heated to 500 °C in a furnace under a 100 mL/min nitrogen flow, with the head kept outside to maintain ELT at room temperature. Once the target pyrolysis temperature was reached (500 °C), the piston introduced the feedstock into the hot reactor, where it was pyrolyzed. The carrier gas transported the produced vapors through the catalyst bed (500 °C) and into a liquid trap submerged in a cooling bath where they condensed to form the pyrolysis oil, while the noncondensable vapors exited the trap and entered the gas collection system through a glass-wool filter. After 15 min, the reactor was cooled to room temperature under a 50 mL/min nitrogen flow. The pyrolysis oil mass was determined from the weight difference of the preweighed trap, while the mass of the gas products was obtained from offline GC analysis of a sample collected in a multilayer foil gas sampling bag. The mass of the total solid products, consisting of pyrolysis residue (char) and coke formed on the catalyst surface, was determined from the weight difference of the preweighed reactor, accounting for the mass of the glass wool and the catalyst. The coked catalyst was recovered from the bottom of the reactor, and its carbon content was determined by elemental analysis.

The PDU consisted of two bubbling-bed reactors connected in series, a continuous screw feedstock feeding system, a vapor condensation system consisting of shell and tube heat exchangers, and a gas meter for volumetric measurement of the produced gases (Figure 2). The temperature in each reactor was maintained at 500 °C and was monitored with three thermocouples placed across its height. For each test, 100 g of ELT was continuously fed at a rate of 5 g/min in the pyrolysis reactor (reactor 1) where it was pyrolyzed in contact with 222 g of inert particles that were fluidized with 1.6 L/min nitrogen through a distributor plate at the reactor base and 2.5 L/min nitrogen through the feeding system. The produced vapors were transferred to reactor 2 that contained varied quantities of catalyst to achieve C/F ratios from 0 to 1, along with an adjusted quantity of inert particles to maintain a constant bed volume across all C/F ratios. Reactor 2 was fluidized with the pyrolysis vapors and an additional 0.8 L/min nitrogen flow through a distributor plate at the reactor base. The upgraded vapors from reactor 2 entered the condensation system where the condensable fraction was collected in a pyrolysis oil vessel. Sintered metal powder filters (10 μm) at the exit of both reactors ensured that no solids were carried over. A dry gas meter was used to measure the volume of noncondensable gases exiting the condensation system. After 20 min, the feeding was stopped, and purging with nitrogen continued for 15 min for the complete stripping of the pyrolysis vapors. The fluidizing gas was then switched to air, and the temperature of reactors 1 and 2 was increased to 650 and 590 °C, respectively, to combust the char in reactor 1 and the coke in reactor 2. During this stage, the condensation system was bypassed, and the flue gas was routed directly to the gas sampling slipstream and the gas meter.

**Figure 2:**
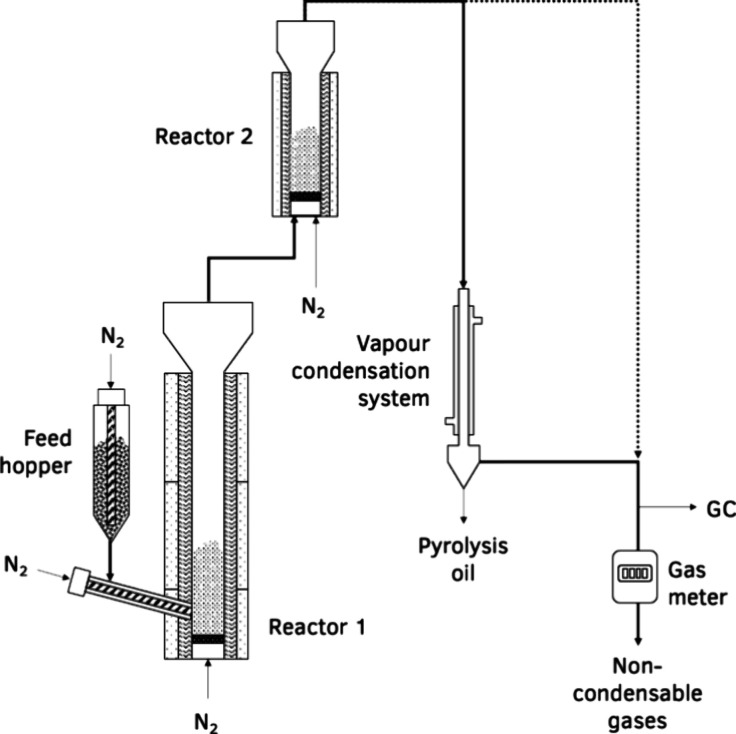
Schematic representation of the continuous PDU equipped with two bubbling-bed reactors connected in series.

The pyrolysis oil was determined by direct weighing, while the gas products were determined by offline GC analysis of a slipstream collected in a multilayer foil gas sampling bag for the duration of each test. The total solid products (char + coke) were determined from the carbon mass in the CO_2_ and CO generated during the combustion stage, quantified by GC analysis of the flue gas.

The residence time of the pyrolysis vapors was estimated according to eq 1:

$$\mathrm{residence}\;\mathrm{time}=\frac{V}{{\mathrm{V̇}}_{\mathrm{vapors}}}$$ 1

were *V̇*_vapors_ was the volumetric flow rate of the pyrolysis vapors, including the carrier gas, and *V* was the volume of the reactor or of the catalyst bed. The residence time in the bench-scale reactor and the PDU was estimated at 7 and 14.5 s, respectively. The contact time between the vapors and the catalyst was estimated at 0.8 s in the bench-scale reactor and 0.9 s in the PDU.

The product yields were calculated according to eq 2:

$${\mathrm{yield}}_{i}=\frac{m_{\mathrm{product},i}}{m_{\mathrm{feedstock}}}\times 100\%$$ 2

were *m*_product,*i*_ was the mass of the pyrolysis product *i* and *m*_feedstock_ was the mass of the feedstock. The mass balance was calculated by summing the yields of oil, solid, and gas products, achieving a closure of 91–98% in both experimental configurations. All yields were normalized to 100% mass balance. Each test point was repeated at least two times, and the average value of the trials is reported.

Catalytic conversion was calculated according to eq 3:

$${\mathrm{conversion}}_{\mathrm{cat},i}=\frac{{\mathrm{HVOilYield}}_{\mathrm{th}}-{\mathrm{HVOilYield}}_{\mathrm{cat},i}}{{\mathrm{HVOilYield}}_{\mathrm{th}}}\times 100\%$$ 3

where HVOilYield_th_ was the heavy oil fraction yield obtained from thermal pyrolysis and HVOilYield_cat,*i*_ was the heavy oil fraction yield obtained from catalytic pyrolysis with catalyst *i*. Conversion expressed the fraction of heavy oil components in the pyrolysis vapors that was converted to other products due to the catalyst’s effect, and it was a direct indication of the catalyst’s activity. This definition followed standard practice as it was analogous to that used in the fluid catalytic cracking of vacuum gas oil, where conversion is defined as the mass or volume fraction of the vacuum gas oil that is converted to lighter products.

The selectivity of each catalyst toward BTX was calculated according to eq 4:

$${\mathrm{BTXSelectivity}}_{\mathrm{cat},i}=\frac{{\mathrm{BTXyield}}_{\mathrm{cat},i}-{\mathrm{BTXyield}}_{\mathrm{th}}}{{\mathrm{conversion}}_{\mathrm{cat},i}}\times 100\%$$ 4

where BTXyield_cat,*i*_ was the BTX yield obtained with catalyst *i*, BTXyield_th_ was the BTX yield obtained from thermal pyrolysis, and conversion_cat,*i*_ was the conversion of heavy oil components (eq 3) achieved with catalyst *i*. This approach provided a basis for comparing different catalysts by normalizing the net BTX yield to the amount of converted heavy oil components.

### 2.4 Analytical Methods

The moisture content of the ELT feedstock was determined by the weight difference of a sample after drying at 105 °C overnight, while the ash content was determined by the solid residue of a sample after combustion in a furnace at 650 °C for 3 h in contact with air. Thermogravimetric analysis was used to characterize the thermal behavior of the ELT feedstock by measuring its mass change from room temperature to 700 °C (5 °C/min) under a nitrogen flow (50 cm^3^/min) using a NETZSCH thermogravimetric analyzer.

Elemental carbon and hydrogen content was determined by complete combustion and infrared (IR) analysis of the produced CO_2_ and H_2_O using a LECO Corporation 628 Series elemental analyzer according to ASTM D5291. Elemental sulfur, zinc, calcium, iron, and potassium content was determined by energy-dispersive X-ray fluorescence using a Petra MAX benchtop spectrometer operated with a 50 kV, 2 mA X-ray tube, a doubly curved crystal optic, and a high-resolution silicon drift detector. Spectra were collected for 300 s, and matrix effects were corrected with methods implemented in the instrument software (Petra Series v4.2). Oxygen content was determined by the difference, according to the formula oxygen% = 100% – ash% – carbon% – hydrogen% – sulfur%.

The composition of the pyrolysis gases (CO, CO_2_, H_2_, and C_1_–C_4_ hydrocarbons) was determined by gas chromatography (GC) using a Hewlett-Packard 5890 Series II gas chromatograph equipped with four columns (precolumn: OV-101; columns: Porapak N, molecular sieve 5 Å, and Rt-Qplot 30 m × 0.53 mm ID), a thermal conductivity detector (TCD), and a flame ionization detector (FID).

The naphtha (C_5_–216 °C), middle distillate (216–343 °C), and heavy (>343 °C) fractions in the pyrolysis oils were determined by simulated distillation analysis according to ASTM D-6352 using an Agilent 6890N gas chromatograph equipped with a programmable temperature vaporization injector, an Analytical Controls SIMDIS HT 750 column (5 m × 0.53 mm × 0.09 μm), and an FID. The boiling points were calibrated with standard mixtures of light (C_5_–C_28_) and heavy (C_30_–C_120_) paraffins.

Benzene, toluene and xylene in the pyrolysis oils were determined by detailed hydrocarbon analysis (DHA) according to ASTM D-6733 using an Agilent 6890N gas chromatograph equipped with a programmable temperature vaporization injector, a Restek Rtx-DHA-50 column (50 m × 0.2 mm × 0.5 μm), and an FID. Identification and quantification of the hydrocarbons were carried out using the Carburane DHA software.

The total MAH and PAH in the pyrolysis oils were determined by high-performance liquid chromatography (HPLC) according to EN 12916 using an Agilent 1100 Series HPLC system equipped with two Agilent LiChrospher 100 NH2 5 μm, 4.0 × 250 mm columns and a refractive index detector. The quantification of the MAH, diaromatic, and tri+-aromatic hydrocarbons was based on calibration with solutions of *o-*xylene, fluorene, and phenanthrene, respectively, according to the standard method.

Sulfur speciation in pyrolysis oil samples was performed by gas chromatography using an Agilent 8890 gas chromatograph coupled to an Agilent 8355 sulfur chemiluminescence detector featuring a dual plasma burner. Peaks were identified by matching their retention times to those of sulfur compound standards and were classified into five groups: nonthiophenic compounds, thiophenes, benzothiophenes, dibenzothiophenes, and heavier sulfur compounds.

The surface and pore properties of the catalysts were determined by nitrogen adsorption–desorption at −196 °C in a Micromeritics TriStar II Plus porosimeter after outgassing under a vacuum at 300 °C for 16 h. The Brunauer–Emmett–Teller (BET) method^48^ was applied to calculate the total surface area, while the t-plot method^49^ was used to determine the micropore surface area and volume. The total pore volume was determined by the volume of physisorbed nitrogen at a *P*/*P*_0_ = 0.99.

The number and type of acid sites of the zeolite-based catalysts were determined via transmission Fourier transform IR spectroscopy using a Thermo Scientific Nicolet 5700 spectrometer combined with *in situ* pyridine adsorption.^50^ The Bro̷nsted and Lewis acid sites were distinguished by the 1545 cm^–1^ (pyridinium ions) and 1450 cm^–1^ (pyridine coordinated to Lewis acid sites) peaks, respectively, and were quantified using the Lambert–Beer law normalized by the catalyst wafer thickness and by adopting the molar extinction coefficients proposed by Emeis.^51^

The Ni and Co content of the zeolite-based catalysts was determined by inductively coupled plasma–atomic emission spectroscopy (ICP-AES) using a 4300 DV PerkinElmer Optima spectrometer after digestion in acid.

XRD spectra were obtained using a Siemens Diffractometer D5000 equipped with Cu K X-ray radiation and a curved crystal graphite monochromator operating at 45 kV and 100 mA. Counts were accumulated in the range of 5–85° 2θ every 0.02° 2θ with a counting time of 2 s per step.

Temperature-programmed reduction with hydrogen (TPR-H_2_) was conducted using 100 mg of catalyst sample loaded into a quartz fixed-bed reactor connected to a mass spectrometer (MS). Prior to reduction, the sample was pretreated in a flow of pure He, heating from room temperature (RT) to 300 °C at a rate of 10 °C/min and maintaining this temperature for 1 h. Following pretreatment, the sample was cooled to RT and then heated to 800 °C at 10 °C/min under a 5 vol % H_2_/He mixture. Hydrogen consumption was monitored continuously via MS by tracking the H_2_ signal at *m*/*z* = 2.

## 3 Results and Discussion

### 3.1 ELT Feedstock Characterization

The physicochemical properties of the ELT feedstock are presented in Table 1. The feedstock exhibited a high carbon content, indicating the predominance of elastomers and carbon black and the lack of steel reinforcements found in original tires. The sulfur and ash contents were within the typical ranges for steel-free waste tire feedstocks, at 1–2 wt % for sulfur and 3–20 wt % for ash.^52^ Additionally, the ELT feedstock contained notable amounts of Zn owing to its role as an activation agent in vulcanization, while elements such as Ca, K, and Fe were present at significantly lower concentrations.

**Table 1:** Physicochemical Properties of the ELT Feedstock Used in This Work

feedstock	ELT
supplier	Michelin
description	granules of multibrand all-tire
particle size, mm	0.2–0.8
moisture, wt %	0.5
ash, wt %	6.6
C, wt %	81.9
H, wt %	7.7
S, wt %	1.6
O, wt %	2.2
Zn, ppm	20,852
Ca, ppm	907
K, ppm	598
Fe, ppm	376

The differential weight loss curve obtained from the thermogravimetric analysis of the ELT is presented in Figure 3. Volatilization was initiated slightly at ca. 215 °C and ceased at ca. 500 °C. A major decomposition peak was observed ca. 372 °C, which can be attributed to the decomposition of the natural rubber (NR) component of the ELT.^4,53^ The shoulder observed at ca. 415 °C can be attributed to the decomposition of the butadiene rubber (BR) and styrene–butadiene rubber (SBR) components.^4,53^ This shape of differential weight loss curve, i.e., main peak in the natural rubber decomposition range with a shoulder in the BR/SBR decomposition range, indicated that the relative content of natural rubber was higher than that of the BR/SBR, a composition that is closer to truck vehicle ELTs.^4,54,55^

**Figure 3:**
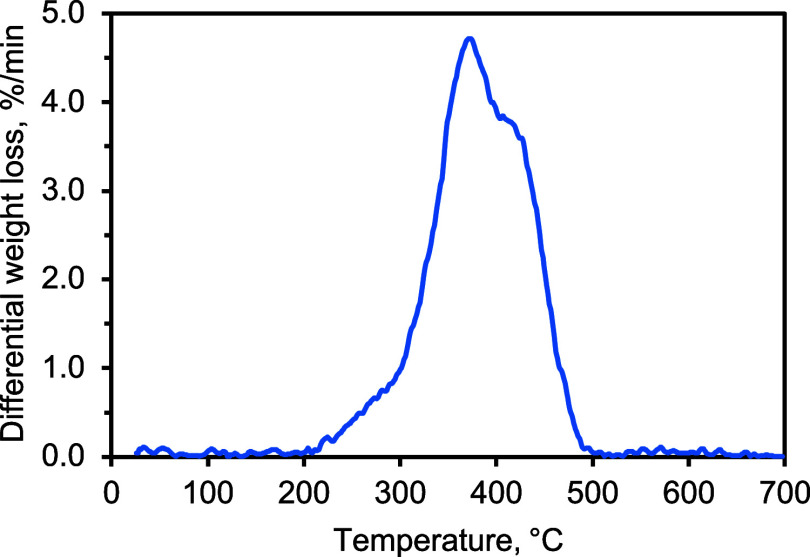
Differential weight loss curve obtained from the thermogravimetric analysis of the ELT feedstock.

### 3.2 Catalyst Characterization

The physicochemical properties of the catalysts used are listed in Table 2. The equilibrium FCC catalyst USY was selected because of the reported high ELT-vapor-cracking activity of Y zeolite,^19^ the main active component of FCC catalysts. Equilibrium catalysts offer a cost-effective alternative to fresh FCC catalysts, while their repurposing in the catalytic pyrolysis of ELTs creates a value-added application for this refinery waste stream. Ni/USY was also an equilibrium FCC catalyst but exhibited significant Ni poisoning (1.6 wt %), and it was selected because of the known dehydrogenation activity of Ni. While Ni-catalyzed dehydrogenation is detrimental in FCC processes,^56^ it could be advantageous to maximize aromatic hydrocarbon formation. ZSM-5 was selected for the shape selectivity of its ZSM-5 zeolite phase that limits coke formation and selectively promotes BTX formation.^26,57^ Co/ZSM-5 was selected on the basis of a previous work where it increased aromatic hydrocarbon formation compared to its Co-free counterpart.^47^

**Table 2:** Physicochemical Properties of the Catalysts Used in This Work

	USY	Ni/USY	ZSM-5	Co/ZSM-5
Ni, wt %	<0.01	1.6		
Co, wt %				5.5
BET surface area, m^2^/g	188	149	162	159
micropore surface area, m^2^/g	129	114	97	118
total pore volume, cm^3^/g	0.235	0.179	0.135	0.156
micropore volume, cm^3^/g	0.051	0.044	0.039	0.047
Bro̷nsted acid sites, μmol pyridine/g	8.8	10.6	5.6	17.3
Lewis acid sites, μmol pyridine/g	14.4	8.3	3.8	45.9

All catalysts exhibited comparable textural properties, with BET surface areas between 149 and 188 m^2^/g. These values were lower than those of pure synthetic zeolites, as all catalysts in this work were technical catalysts composed of a zeolite phase embedded in a matrix. The catalysts also exhibited comparable acidity levels, with the exception of Co/ZSM-5 that exhibited markedly higher Lewis acidity attributed to its cobalt doping.^47^ Notably, the metal loading on Ni/USY (1.6 wt % Ni) and Co/ZSM-5 (5.5 wt % Co) differed significantly. The aim here was to evaluate the dehydrogenation effect of the metal-loaded catalysts by comparison to their metal-free counterparts, not by comparison to the two different metal-loaded catalysts.

The XRD diffraction patterns, presented in Figure 4, confirmed the presence of Y zeolite (FAU) phases in USY and Ni/USY and ZSM-5 zeolite (MFI) phases in ZSM-5 and Co/ZSM-5. Additionally, reflections attributable to NiO (mainly at 37.2 and 43.2° 2θ) and Co_3_O_4_ (mainly at 31.2, 36.8, and 59.5° 2θ) were detected in the diffraction patterns of Ni/USY and Co/ZSM-5, respectively.

**Figure 4:**
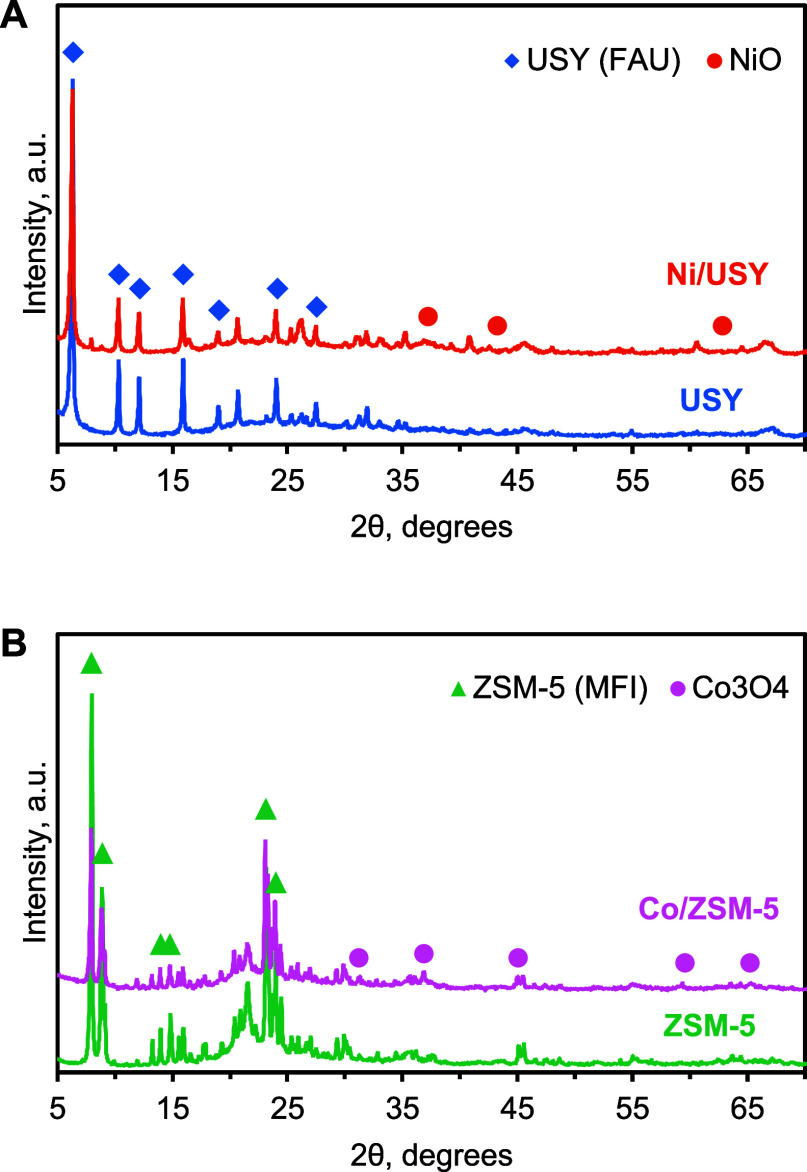
XRD diffractograms of USY, Ni/USY (A) and ZSM-5, Co/ZSM-5 (B) catalysts.

The reducibility of Ni/USY and Co/ZSM-5 was investigated via TPR-H_2_, and the reduction profiles are presented in Figure 5. In the case of Ni/USY, a broad reduction peak was observed starting at 270 °C, with a maximum at 780 °C and a shoulder around 490 °C. This broad reduction profile was initially related to the reduction of bulk NiO species (shoulder at 490 °C) on the outer catalyst surface with weak Ni-support interaction, while the higher temperature peak was attributed to the reduction of Ni species strongly interacting with the zeolite phase.^58,59^ On the other hand, the reduction profile of Co/ZSM-5 exhibited two main reduction peaks; the first was observed at 300–500 °C, with a maximum at 360 °C, and the second was observed at temperatures higher than 500 °C, with a maximum at 780 °C. The first peak was attributed to the reduction of Co_3_O_4_ species to CoO, and the second was attributed to the reduction of CoO. The high temperature area of the second peak can be also attributed to the reduction of cobalt oxide species strongly interacting with the support.^60^

**Figure 5:**
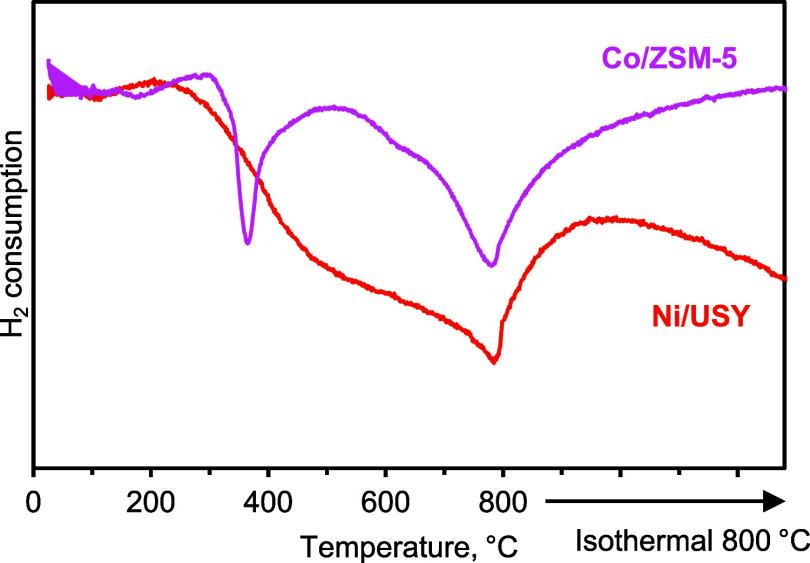
TPR profiles of the Ni/USY and Co/ZSM-5 catalysts.

### 3.3 Bench-Scale Catalyst Screening

#### 3.3.1 Product Yields

The catalyst screening tests were carried out at 500 °C (pyrolysis and catalytic upgrading). This temperature was selected based on the thermogravimetric analysis of the feedstock, which indicated that complete thermal decomposition was achieved at ca. 500 °C (Figure 3). For reference, pyrolysis without a catalyst (thermal) was also carried out at 500 °C. The results obtained are presented in Table 3.

**Table 3:** Results from the ELT Thermal and Catalytic Pyrolysis Tests in the Bench-Scale Fixed-Bed Reactor at 500 °C (C/F = 3 in the Catalytic Tests)

	no catalyst	USY	Ni/USY	ZSM-5	Co/ZSM-5
**Yields of main pyrolysis products,**wt % on dry feedstock					
pyrolysis oil	57.0	45.9	48.3	49.6	49.1
gases	4.4	12.0	10.5	12.6	11.7
char	38.6	37.5	35.7	36.3	35.4
coke	0.0	4.6	5.5	1.5	3.8
**Yields of individual gas products,**wt % on dry feedstock					
CO_*x*_	0.7	0.6	0.6	0.7	0.6
H_2_	0.0	0.1	0.5	0.1	0.4
C_1_	0.4	1.2	1.0	0.5	0.6
C_2_	0.5	1.2	1.1	2.1	2.0
C_3_	0.6	3.3	2.9	5.7	5.0
C_4_	2.2	5.6	4.4	3.5	3.1
**Yields of pyrolysis oil fractions,**wt % on dry feedstock					
naphtha (C_5_–216 °C)	17.7	30.5	28.7	24.5	19.2
middle distillate (216–343 °C)	17.5	12.4	15.9	15.5	18.3
heavy (>343 °C)	21.8	3.0	3.7	9.6	11.6
**Conversion, %** ^a^					
heavy fraction conversion	n.a.^b^	86.2	83.0	56.0	46.8
**Yields of BTX,**wt % on dry feedstock					
benzene	0.0	0.3	0.2	0.5	0.4
toluene	0.4	4.7	3.9	3.5	3.5
xylene	1.2	5.6	5.0	5.1	4.7
**total BTX**	**1.6**	**10.6**	**9.1**	**9.1**	**8.6**
**BTX** **selectivity, %** ^c^					
BTX selectivity	n.a.^b^	10.4	9.0	13.4	15.0
**Yields of total aromatic hydrocarbons,**wt % on dry feedstock					
MAH	n.d.^d^	23.3	24.2	25.7	25.6
PAH	n.d.^d^	14.7	17.8	8.8	10.0
**total aromatic hydrocarbons**	**n.d.^d^**	**38.0**	**42.0**	**34.5**	**35.6**
**Pyrolysis oil elemental composition**					
C, wt %	81.3	88.4	88.9	87.7	88.4
H, wt %	11.0	9.8	10.0	10.4	10.4
C/H molar ratio	0.62	0.75	0.74	0.70	0.71

a Calculated according to eq 3.

b n.a. = not applicable as conversion and selectivity were calculated based on the thermal pyrolysis results.

c Calculated according to eq 4.

d n.d. = not determined as oil samples with high final boiling points were not suitable for HPLC analysis.

Thermal pyrolysis of the ELT yielded 57.0 wt % oil, 4.4 wt % gases, and 38.6 wt % solid residue (char). These yields were within the ranges typically reported for the thermal pyrolysis of ELTs at 500 °C.^52^

In the catalytic pyrolysis tests, catalytic cracking reactions reduced the oil yield to 45.9–55.8 wt %, depending on the catalyst used, and increased the gas product yield to 5.9–12.6 wt %. Char yields appeared lower in the catalytic pyrolysis tests (35.4–37.5 wt %). However, because there was no contact between the feedstock and the catalyst in the reactor, this reduction was attributed to experimental variability related to the handling and accurate weighing of small quantities of solid samples rather than to changes in feedstock volatilization. Catalytic pyrolysis also resulted in the formation of solid deposits on the surface of the catalysts (coke), whose yield was determined via analysis of the recovered catalyst samples. The lowest coke yield (1.5 wt %) was obtained with the ZSM-5, while a higher coke yield (4.6 wt %) was obtained with USY. The metal-loaded Co/ZSM-5 and Ni/USY gave higher coke yields (3.8 and 5.5 wt %, respectively) compared with their metal-free counterparts, which could be linked to dehydrogenation reactions catalyzed on the metal sites contributing to the formation of more condensed aromatic structures and coke. The comparatively higher hydrogen yields obtained with Ni/USY and Co/ZSM-5 were evidence of their enhanced dehydrogenation activity (Table 3). A more detailed discussion of the reaction pathways is provided in a later section. The main gas products from the thermal pyrolysis of the ELT were C_4_ hydrocarbons, attributed to the cracking of the BR and SBR polymer chain and the release of the 1,3-butadiene monomer.^28^ Catalytic upgrading of the pyrolysis vapors resulted in a substantial increase of the C_1_–C_4_ gases, which was attributed to the enhanced cracking of rubber oligomers and smaller intermediates in the primary vapors. USY and Ni/USY were more selective toward C_1_ and C_4_ gases, while ZSM-5 and Co/ZSM-5 favored C_2_ and C_3_ gases. Similar gas product selectivities for Y and ZSM-5 catalysts applied in ELT pyrolysis were reported by Williams and Brindle.^18^

#### 3.3.2 Pyrolysis Oil Composition

The composition of the pyrolysis oils in terms of naphtha range (boiling point <216 °C), middle distillate range (boiling point 216–343 °C), and heavier (boiling point >343 °C) compounds was determined by simulated distillation analysis. The oil obtained from thermal pyrolysis was heavier than those obtained from catalytic pyrolysis. The heavy oil yield from thermal pyrolysis was 21.8 wt %, while the naphtha fraction yield was 17.7 wt %. Catalytic pyrolysis significantly decreased the heavy oil yield to 3.0–14.4 wt % and increased the naphtha fraction yield to 19.2–30.5 wt %. USY and Ni/USY achieved the highest heavy-oil-fraction conversion (eq 3), while ZSM-5 and Co/ZSM-5 were less active.

The BTX yield from thermal pyrolysis was 1.6 wt % but increased with catalytic pyrolysis up to a maximum of 10.6 wt % (Table 3). Although the ZSM-5 catalysts achieved moderate heavy-oil-fraction conversion, they produced relatively high BTX yields, resulting in markedly higher BTX selectivity compared to the USY catalysts. Accordingly, slightly higher MAH yields were obtained with the ZSM-5 catalysts, while the USY catalysts yielded more PAHs. Comparing the metal-loaded and metal-free catalysts, the main difference was observed in the relative MAH vs PAH yields; both Ni/USY and Co/ZSM-5 gave higher yields of PAHs compared to their metal-free counterparts. The highest total aromatic hydrocarbon yield was obtained with Ni/USY (42.0 wt %), notably higher that that obtained with the metal-free USY (38.0 wt %). Taking into account the lower conversion achieved with Ni/USY, it was indicated that its selectivity toward aromatic hydrocarbons was superior to that of the metal-free USY.

The total aromatic hydrocarbon yield from thermal pyrolysis could not be determined, as the thermal pyrolysis oil contained high-boiling-point compounds that rendered it unsuitable for analysis via HPLC. Instead, the molar C/H ratio was used as an indicator of aromaticity, with higher values reflecting greater aromatic content. The C/H ratio increased from 0.62 in thermal pyrolysis to 0.70–0.75 in the catalytic tests, indicating an increase in the aromaticity. The highest C/H ratios were obtained with USY and Ni/USY. The metal-loaded and metal-free zeolite catalysts could not be differentiated based on the C/H ratio as their differences were minor and within experimental error.

Summarizing the catalyst screening results, the USY catalysts demonstrated high activity for the conversion of heavy compounds in the ELT pyrolysis vapors and maximized the total aromatic hydrocarbon yield. The ZSM-5 catalysts were comparatively less active but demonstrated superior selectivity toward BTX hydrocarbons. The differences in conversion and selectivity observed between the USY and ZSM-5 catalysts were related to the pore size of their respective zeolite phase; the pore diameter of Y (FAU) zeolite (7.4 Å) is larger than that of ZSM-5 (MFI) zeolite (5.6 Å).^61^ As a result, more vapor molecules could readily access the USY and Ni/USY pores, achieving higher conversion. On the other hand, ZSM-5 and Co/ZSM-5 restricted aromatic growth in their narrow pore network, favoring the formation of BTX and MAH over PAH. The pore size effect is discussed in greater detail in a later section. The presence of Ni or Co on the catalysts enhanced the dehydrogenation reactions; Ni/USY was less active for the conversion of heavy compounds compared to USY but demonstrated enhanced aromatization activity that resulted in higher PAH and total aromatic hydrocarbon yields. A similar effect was observed with Co/ZSM-5.

### 3.4 Catalyst Testing in the Continuous PDU

Based on the results of the catalyst screening, the USY, Ni/USY, and Co/ZSM-5 catalysts were selected for testing in the continuous PDU. USY and Ni/USY were selected because of their high activity and high aromatic hydrocarbon yields. Co/ZSM-5 was selected because of its high selectivity toward BTX. Additionally, the inclusion of the Co/ZSM-5 aimed to verify the differences observed between the USY and the ZSM-5 catalysts during the catalyst screening. Each catalyst was tested at three different C/F ratios by changing the amount of catalyst in the catalytic reactor. For reference, thermal pyrolysis tests were also performed during which the catalytic reactor was filled with inert material particles instead of being bypassed to maintain the same vapor residence time. The temperatures of both reactors were kept at 500 °C to maintain the same pyrolysis and catalytic upgrading temperatures used in the bench-scale tests. The results are presented in Table 4.

**Table 4:** Results from the ELT Thermal and Catalytic Pyrolysis Tests in Continuous PDU (Pyrolysis and Catalytic Upgrading at 500 °C)

	no catalyst	USY			Ni/USY			Co/ZSM-5		
	C/F = 0	C/F = 0.5	C/F = 0.75	C/F = 1	C/F = 0.25	C/F = 0.5	C/F = 1	C/F = 0.25	C/F = 0.5	C/F = 1
**Yields of main pyrolysis products,**wt % on dry feedstock										
pyrolysis oil	54.6	48.7	47.4	46.6	51.8	51.3	48.0	52.4	51.3	47.3
gases	6.5	9.3	11.6	10.5	8.0	7.9	9.1	8.2	9.1	10.4
solid products (char + coke)	38.9	42.0	41.0	42.9	40.2	40.8	42.9	39.4	39.6	42.3
**Yields of individual gas products,**wt % on dry feedstock										
CO_*x*_	1.2	1.3	1.4	1.3	1.4	1.1	1.2	1.6	1.4	1.4
H_2_	0.0	0.1	0.1	0.1	0.2	0.2	0.3	0.1	0.1	0.2
C_1_	0.9	0.9	1.3	1.2	0.8	0.8	0.8	0.7	0.9	0.9
C_2_	1.2	1.0	1.4	1.3	1.1	1.0	1.0	1.2	1.5	1.6
C_3_	1.2	2.1	2.8	2.8	1.6	1.7	2.1	2.2	2.8	3.8
C_4_	2.0	3.9	4.6	3.8	2.9	3.1	3.7	2.4	2.4	2.5
**Yields of pyrolysis oil fractions,**wt % on dry feedstock										
naphtha (C_5_–216 °C)	22.7	24.9	26.6	27.0	24.5	25.3	25.1	21.4	20.8	20.0
middle distillate (216–343 °C)	19.0	18.4	16.1	15.6	19.7	19.4	17.7	18.6	18.7	17.5
heavy (>343 °C)	12.9	5.4	4.7	4.0	7.6	6.6	5.2	12.4	11.8	9.8
**Conversion, %** ^a^										
heavy fraction conversion	n.a.^b^	58.1	63.6	69.0	41.1	48.8	59.7	3.9	8.5	24.0
**Yields of BTX,**wt % on dry feedstock										
benzene	0.1	0.1	0.1	0.1	0.1	0.1	0.1	0.1	0.1	0.2
toluene	1.1	2.0	3.1	3.1	1.1	1.2	1.7	1.2	1.6	2.2
xylene	3.0	3.6	5.0	5.1	3.1	3.3	3.8	3.1	3.6	3.9
**total BTX**	4.2	5.7	8.2	8.3	4.3	4.6	5.6	4.4	5.3	6.3
**BTX** **selectivity, %** ^c^										
BTX selectivity	n.a.^b^	2.6	6.3	5.9	0.2	0.8	2.3	5.2	12.9	8.7
**Yields of total aromatic hydrocarbons,**wt % on dry feedstock										
MAH	n.d.^d^	24.7	24.0	25.2	n.d.^d^	25.4	24.8	n.d.^d^	n.d.^d^	24.5
PAH	n.d.^d^	13.5	14.5	13.8	n.d.^d^	14.2	17.1	n.d.^d^	n.d.^d^	10.0
**total aromatic hydrocarbons**	n.d.^d^	38.2	38.5	39.1	n.d.^d^	39.6	41.9	n.d.^d^	n.d.^d^	34.5
**Pyrolysis oil elemental composition**										
C, wt %	88.0	89.5	89.9	89.5	88.6	89.0	89.9	89.6	89.1	88.6
H, wt %	10.5	9.7	9.6	9.7	10.0	9.9	9.5	10.3	10.2	10.0
S, wt %	0.72	0.63	0.61	0.60	0.66	0.62	0.61	0.70	0.68	0.64
C/H ratio	0.70	0.77	0.78	0.77	0.74	0.75	0.79	0.72	0.73	0.74

a Calculated according to eq 3.

b n.a. = not applicable as conversion and selectivity were calculated based on the thermal pyrolysis results.

c Calculated according to eq 4.

d n.d. = not determined as oil samples with high final boiling points were not suitable for HPLC analysis.

#### 3.4.1 Product Yields

The yields of pyrolysis oil, gas products, and solid products obtained from the thermal and catalytic pyrolysis of the ELT in the PDU are presented as a function of the C/F ratio in Figure 6. Thermal pyrolysis yielded 54.6 wt % oil, 6.5 wt % gases, and ca. 38.9 wt % char. The product distribution was similar to that obtained from the bench-scale tests and remained within the typical values reported for the pyrolysis of ELTs at 500 °C.^52^

**Figure 6:**
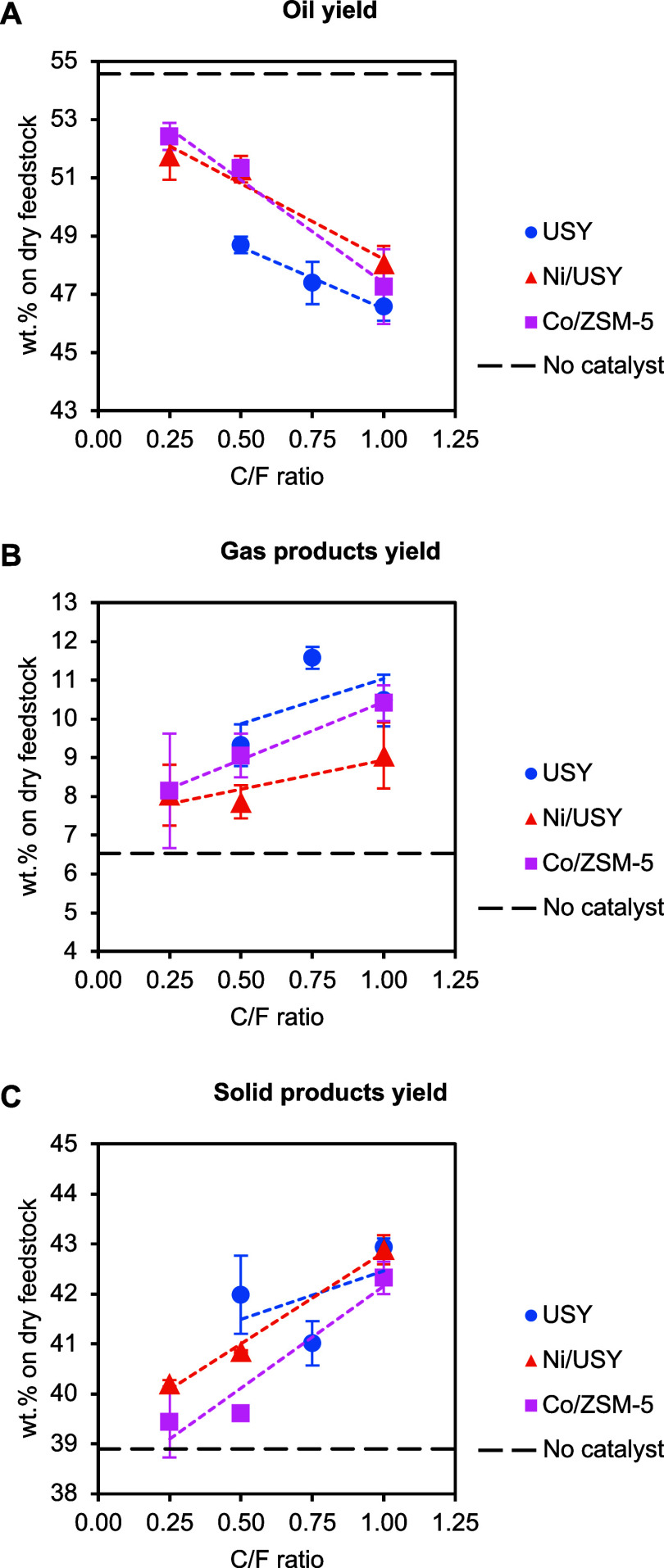
Yields of pyrolysis oil (A), gas products (B), and solid products (C) from the ELT thermal and catalytic pyrolysis tests in the continuous PDU as a function of the C/F ratio (pyrolysis and catalytic upgrading at 500 °C).

As expected, catalytic pyrolysis resulted in a reduction of the oil yield down to a minimum of 46.6 wt % in favor of the gas product yield that increased up to a maximum of 11.6 wt %. An increase of the solid products yield (including both char and coke) was observed as well, attributed to the formation of coke on the catalyst surface. The intensity of these effects correlated to the C/F ratio. USY and Co/ZSM-5 favored gas formation at the expense of the oil yield, while Ni/USY was the least favorable toward gases. The catalytic effect on the individual gas product yields, provided in Table 4, was consistent with the bench-scale tests; the USY catalysts favored C_1_ and C_4_ gases, while Co/ZSM-5 favored C_2_ and C_3_ gases. Furthermore, Ni/USY gave high hydrogen gas yields, confirming the dehydrogenation activity observed in the bench-scale tests.

#### 3.4.2 Pyrolysis Oil Composition

The yields of the naphtha (C_5_–216 °C), middle distillate (216–343 °C), and heavy oil (>343 °C) fractions determined by simulated distillation are presented in Figure 7. Tabulated data are listed in Table 4. The heavy oil fraction yield from thermal pyrolysis was 12.9 wt %. Catalytic pyrolysis with the USY catalysts cracked the heavy ELT vapor compounds and reduced the heavy oil fraction yield mostly in favor of the naphtha fraction. As shown in Figure 8, higher conversion of the heavy fraction was obtained with USY compared to Ni/USY. Furthermore, USY notably increased the naphtha oil fraction yield (Figure 7A), while Ni/USY was comparatively more selective toward middle-distillate-range products (Figure 7B). Compared to the two USY catalysts, a significantly lower conversion of the heavy oil fraction was obtained with Co/ZSM-5 (Figure 8). Notably, Co/ZSM-5 did not increase the yield of either naphtha or middle distillate and instead favored the gas product yield (Figure 6), which was comparatively high considering its low heavy oil fraction conversion activity.

**Figure 7:**
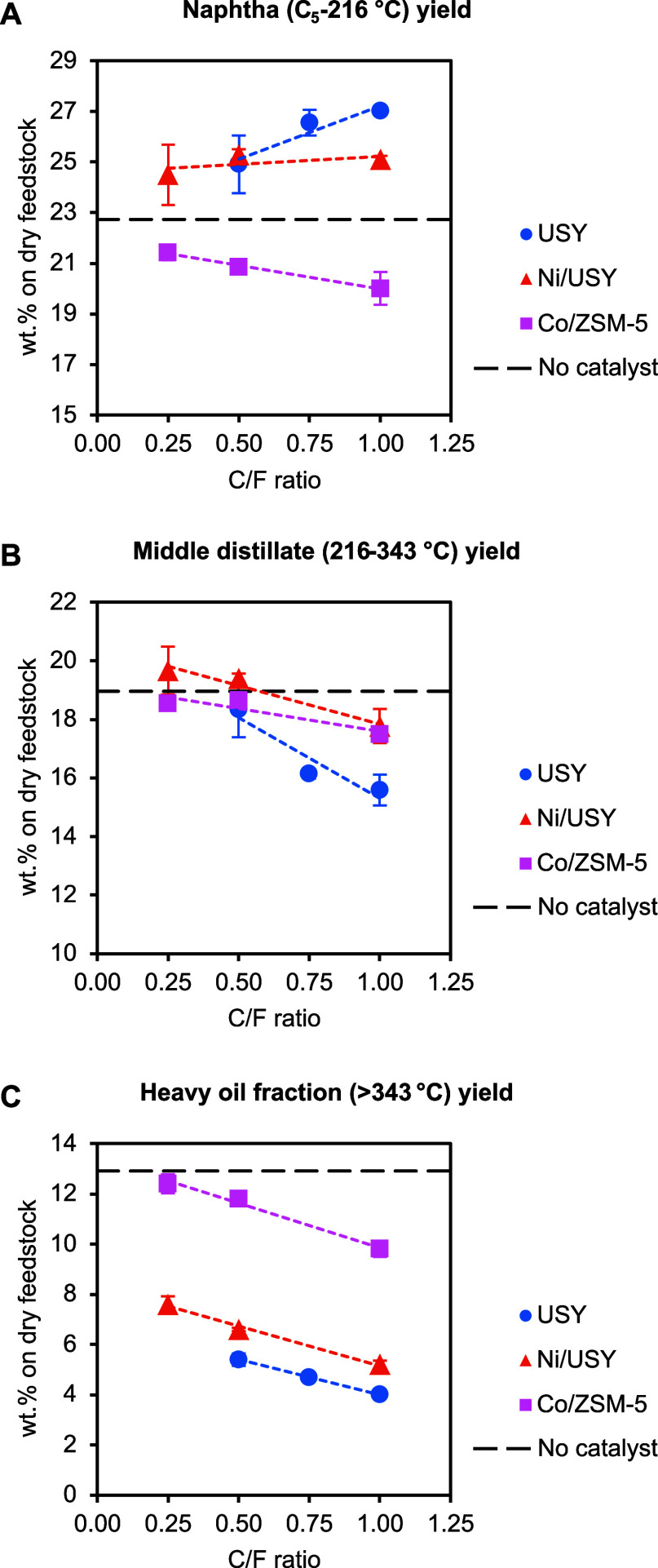
Yields of naphtha (C_5_–216 °C) (A), middle distillate (216–343 °C) (B), and heavy (>343 °C) (C) oil fraction obtained from the ELT thermal and catalytic pyrolysis tests in the continuous PDU as a function of the C/F ratio (pyrolysis and catalytic upgrading at 500 °C).

**Figure 8:**
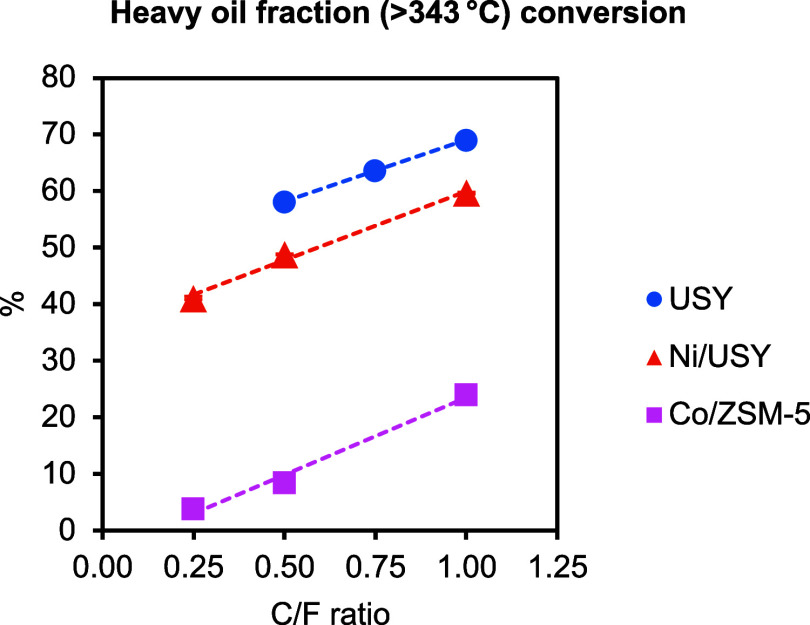
Conversion of the heavy oil fraction (>343 °C) obtained from the ELT catalytic pyrolysis tests in the continuous PDU as a function of the C/F ratio (pyrolysis and catalytic upgrading at 500 °C).

Figure 9 presents the BTX yields and selectivities obtained from the PDU tests. The highest BTX hydrocarbon yield (8.3 wt %) was obtained with the USY catalyst. The BTX yield obtained with Co/ZSM-5 was up to 6.3 wt %, while that obtained with Ni/USY was up to 5.6 wt %. However, Co/ZSM-5 demonstrated the highest selectivity toward BTX hydrocarbons, validating the bench-scale test observations. Ni/USY demonstrated the lowest selectivity to BTX hydrocarbons, also in good agreement with the bench-scale tests.

**Figure 9:**
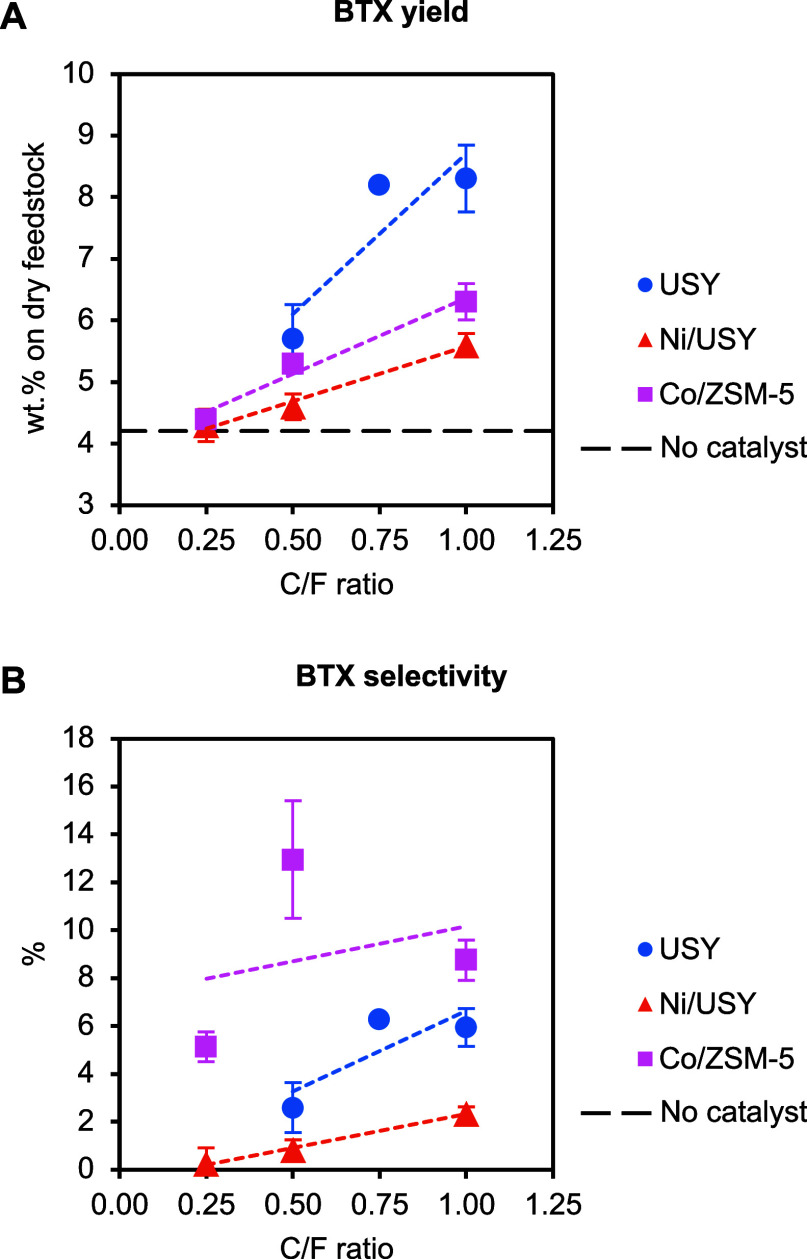
BTX yields (A) and BTX selectivity (B) obtained from the ELT thermal and catalytic pyrolysis tests in the continuous PDU as a function of the C/F ratio (pyrolysis and catalytic upgrading at 500 °C).

The MAH, PAH, and total aromatic hydrocarbon yields obtained from the PDU tests are presented in Figure 10. The highest total aromatic hydrocarbon yield (41.9 wt %) was obtained with Ni/USY at C/F = 1, while total aromatic hydrocarbon yields with USY and Co/ZSM-5 reached up to 39.1 and 34.5 wt %, respectively. Notably, Ni/USY was more selective toward PAH compared to the metal-free USY, attributed to the dehydrogenation effect of Ni. The overall aromaticity of the pyrolysis oils, as reflected by their C/H ratio, increased with catalytic pyrolysis. The oil produced from the thermal pyrolysis of the ELTs exhibited a C/H ratio of 0.70. The highest aromaticity was observed in oils obtained using the Ni/USY (C/F = 0.79) and USY (C/H = 0.78) catalysts at the maximum C/F ratio, while the increase in C/H ratio with the Co/ZSM-5 catalyst was more moderate (C/H = 0.74).

**Figure 10:**
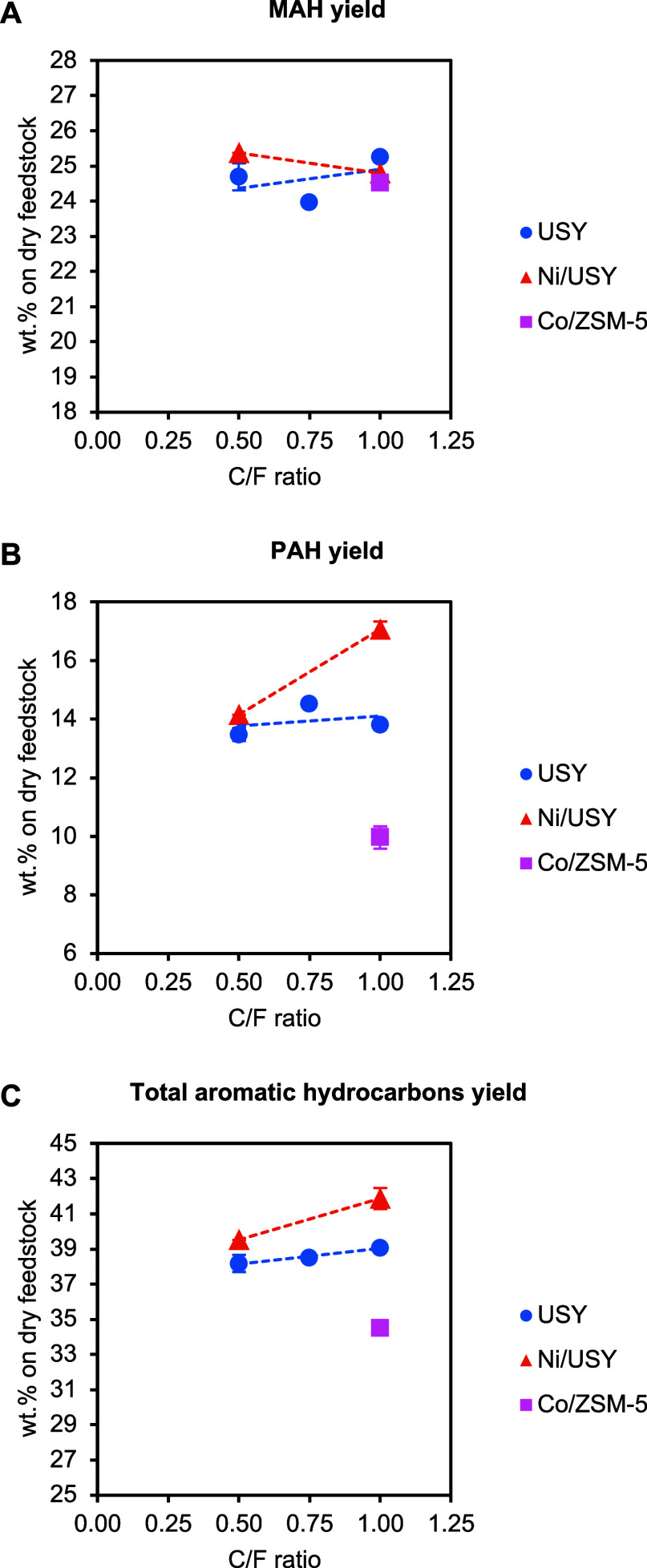
Yields of MAH (A), PAH (B), and total aromatic hydrocarbons (C) obtained from the ELT catalytic pyrolysis tests in the continuous PDU as a function of the C/F ratio (pyrolysis and catalytic upgrading at 500 °C).

The elemental sulfur content in the thermal pyrolysis oil was 0.72 wt %. Catalytic pyrolysis yielded oils with a slightly decreased sulfur content, with higher catalyst loadings favoring lower sulfur. At the highest C/F ratio, sulfur in the pyrolysis oils decreased to 0.60 wt % with USY, 0.61 wt % with Ni/USY, and 0.64 wt % with Co/ZSM-5. Char recovered from the bench-scale tests exhibited a sulfur content of 2.7 wt %, which showed that the majority of sulfur from the ELT feedstock was retained in the char (64%), while 24% was transferred to the thermal pyrolysis oil and, by difference, 11% was transferred to the pyrolysis gases. Catalytic pyrolysis shifted sulfur distribution to a lower 17–19 wt % in the pyrolysis oil and a higher 17–18 wt % in the pyrolysis gases. These distributions were in line with other reports.^62^

Figure 11 presents the sulfur speciation in the pyrolysis oils, which revealed that sulfur was present in the form of thiophenes (Th), benzothiophenes (BzTh), dibenzothiophenes (DiBzTh), and heavier sulfur-containing compounds, with minimal contribution from nonthiophenic species (Non-Th). Catalytic upgrading increased the proportion of thiophenes and dibenzothiophenes and reduced benzothiophenes and heavier sulfur compounds. The effect was comparable for USY and Ni/USY, and it was slightly more moderate for Co/ZSM-5.

**Figure 11:**
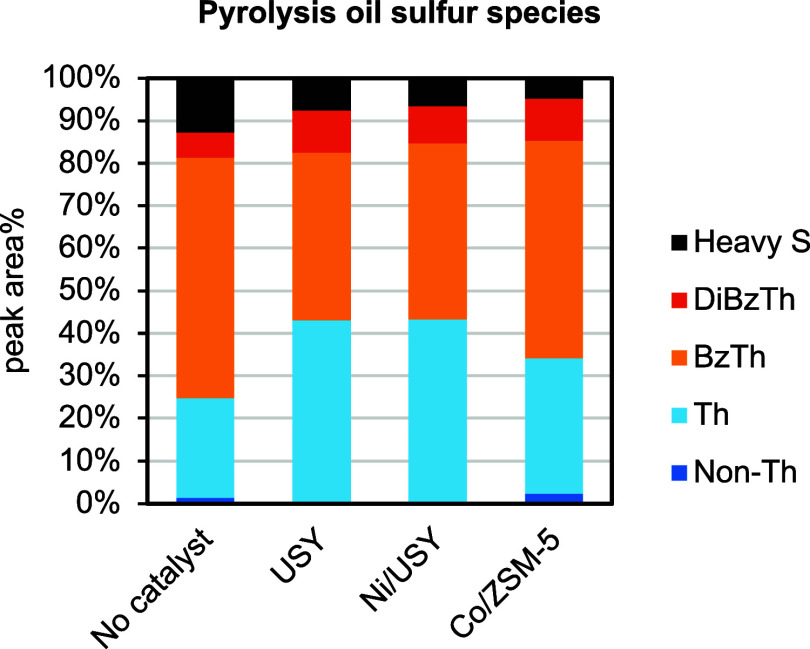
Sulfur speciation of the pyrolysis oils obtained from the ELT thermal and catalytic pyrolysis tests in the continuous PDU (C/F = 1, pyrolysis and catalytic upgrading at 500 °C).

### 3.5 Comparison of Bench-Scale and PDU Results

Through a direct comparison of the data obtained from the PDU and the bench-scale tests, it was observed that the yields of oil, gas, and solids were similar, with some key differences. The oil yield from thermal pyrolysis in the PDU was lower (54.6 wt %) compared to that obtained in the bench-scale tests (57.0 wt %), while the gas product yield was higher (6.5 vs 4.4 wt %). The observed differences were attributed to secondary thermal cracking reactions, facilitated by the roughly 2-fold longer vapor residence time in the PDU (14.5 s) relative to the bench-scale unit (7 s). Different feedstock heating rates that characterize fixed-bed vs fluidized-bed reactors may have also influenced the product distribution. The solid product yield from thermal pyrolysis was similar in both cases (ca. 39 wt %), which was expected considering the identical ELT pyrolysis temperature and the fact that the residence time of the pyrolysis vapors would have no effect on the char in either setup. The difference in the thermal cracking effect between the two setups was also evident from the difference in the heavy oil fraction yield obtained from thermal pyrolysis, which was higher in the bench-scale tests (21.8 wt %) and lower in the PDU (12.9 wt %). Another notable difference between the two setups was that lower catalyst loadings (C/F = 0.25–1) were necessary in the PDU to achieve a catalytic effect comparable to that achieved in the bench-scale tests (C/F = 3). This effect was attributed to improved vapor-catalyst mixing in the bubbling-bed reactor relative to the fixed-bed reactor used in the bench-scale setup. It could further be attributed to the more extensive thermal cracking in the PDU, which facilitated the cracking of large molecules to smaller ones that could more readily access the catalyst active sites.

Importantly, the ranking of the catalysts from the bench-scale catalyst screening in terms of heavy oil fraction conversion activity, yield of different aromatic hydrocarbon species (BTX, MAH, and PAH), yield of total aromatic hydrocarbons, selectivity toward BTX, and capacity to increase the overall oil C/H ratio was validated without deviations in the PDU tests. Furthermore, the maximum yields of the different aromatic hydrocarbon species obtained from the two units were comparable. This was also true for the composition of the pyrolysis oils obtained at high conversion. As shown in Figure 12, a comparison of the hydrocarbon species content in the oils obtained from the bench-scale unit (C/F = 3) and the PDU (C/F = 1) showed remarkable similarities in the levels of MAHs, PAHs, and total aromatic hydrocarbons. The only notable difference was the lower content of BTX in the pyrolysis oils from the PDU. In both cases, the most aromatic pyrolysis oils were obtained with Ni/USY and were composed of 87 wt % aromatic hydrocarbons. The remaining 13 wt % was attributed to olefins, naphthenes, and paraffins, as well as oligomers derived from the thermal decomposition of the ELTs.

**Figure 12:**
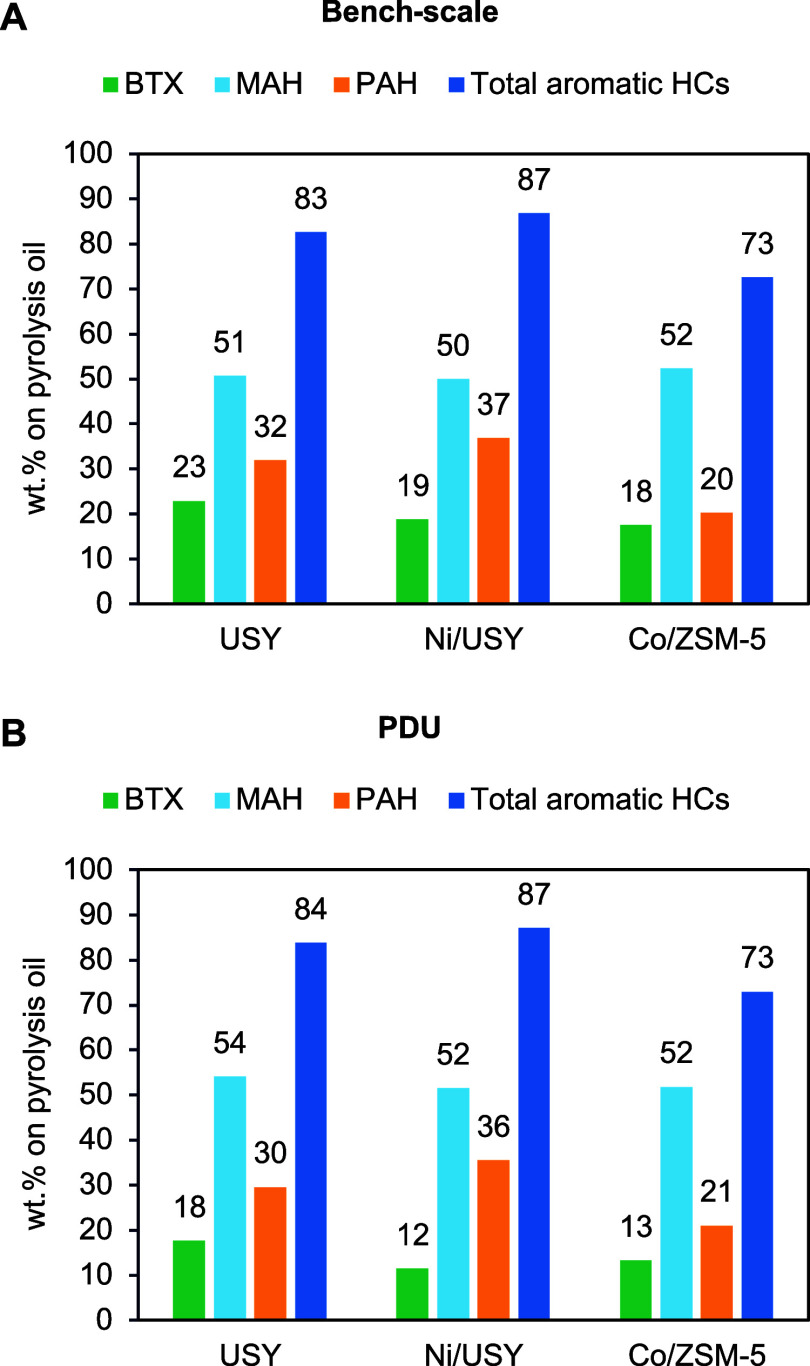
Comparison of the aromatic hydrocarbon species content in the pyrolysis oils obtained from ELT catalytic pyrolysis in (A) the bench-scale fixed-bed reactor (C/F = 3) and (B) the PDU (C/F = 1).

Direct comparison with previous studies is challenging due to differences in reactor designs, catalysts, and analytical methods used by various research groups. However, focusing on reports of catalytic pyrolysis of ELTs carried out in continuous systems at 500 °C provides a useful context. Williams and Brindle^20^ reported pyrolysis oils containing 18.3 wt % BTX using an HY catalyst at a C/F ratio of 1. Olazar et al.^25^ reported oils with 3.5 wt % BTX, 40.5 wt % MAH (including BTX), and 36.7 wt % PAH content, amounting to a total aromatic hydrocarbon content of 77.2 wt %, when using an HY catalyst at a higher C/F ratio of 7.5. In the present study, using equilibrium FCC catalysts at C/F = 1 in the PDU, the maximum BTX content (18 wt %) matched that reported by Williams and Brindle,^20^ while the PAH content (30–36 wt %) was comparable to the findings of Olazar et al.^25^ Notably, the MAH content observed here was higher (52–54 wt %), leading to higher total aromatic hydrocarbon contents of up to 87 wt %. Reports on the catalytic pyrolysis of ELTs with equilibrium FCC catalysts are limited. Salmasi et al.^29^ reported pyrolysis oils with 43.2% relative abundance of aromatic hydrocarbons obtained from the catalytic pyrolysis of polybutadiene rubber with an equilibrium FCC catalyst at C/F = 0.6. Tian et al.^40^ reported pyrolysis oils with a total aromatic hydrocarbon content of ca. 36.4 wt % using an equilibrium FCC catalyst mixed with ELTs at C/F = 0.03.

### 3.6 Reaction Pathways

Figure 13 illustrates the proposed thermal degradation and catalytic conversion pathways of ELTs. These pathways, as discussed in detail below, are based on established pyrolysis mechanisms for organic substrates, known catalytic effects of zeolites and transition metals, catalytic pyrolysis data from this study, and previously reported insights into the thermal conversion of NR, BR, SBR, and ELTs.

**Figure 13:**
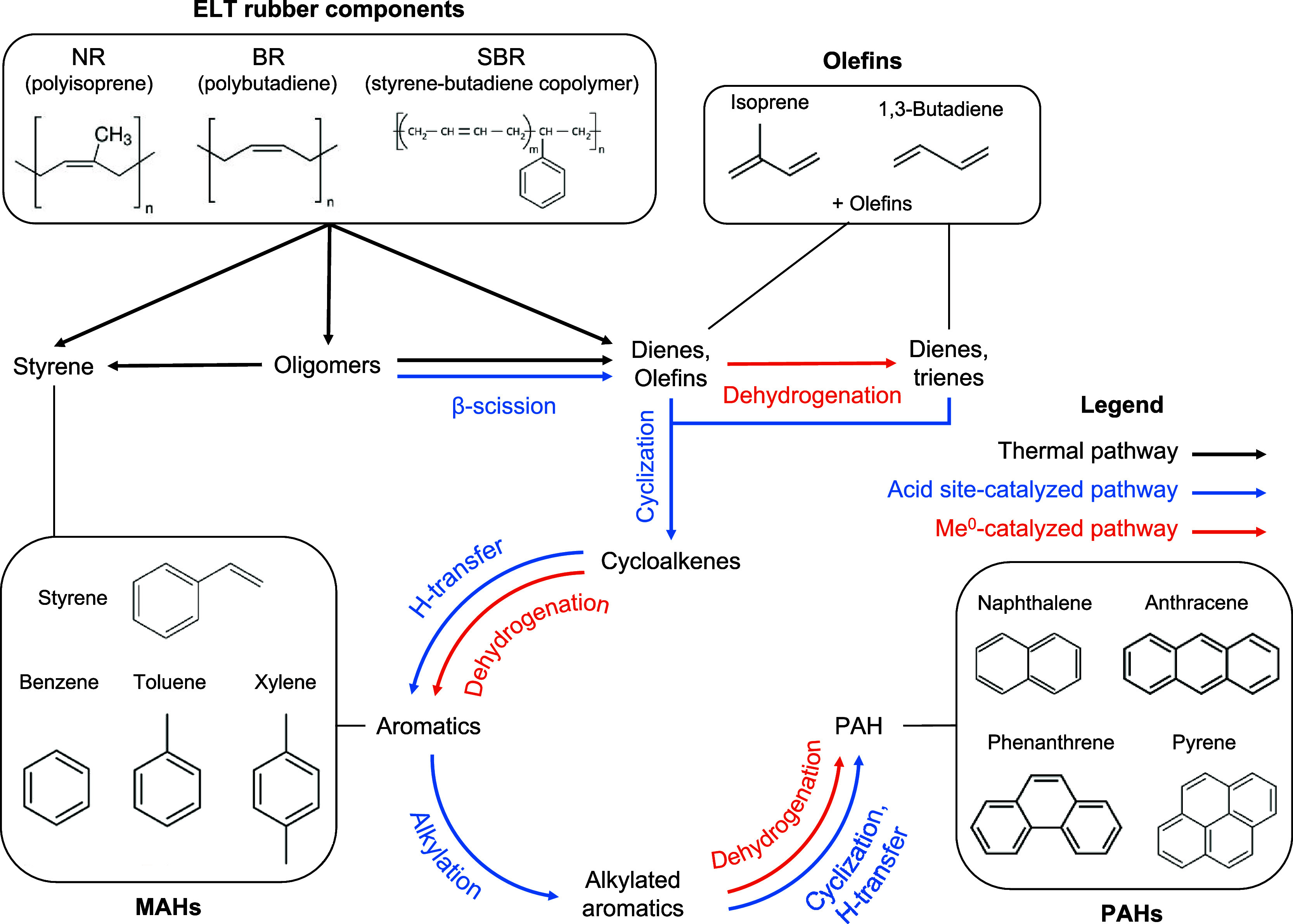
Reaction pathways illustrating the thermal degradation of ELT polymers and the catalytic transformation of the resulting products by zeolite acid sites and Me^0^ sites.

Thermal cracking of the ELT components NR (polyisoprene) and BR/SBR (polybutadiene/styrene–butadiene copolymer) was initiated by the homolytic scission of the C–C bonds in the polymer chains forming free radicals^28,63,64^ and propagated by free-radical-induced chain reactions, yielding rubber monomers (styrene, isoprene, and 1,3-butadiene) and other olefins, commonly observed as products in thermal ELT and rubber pyrolysis studies.^28,63,65–68^ The notable presence of compounds with boiling point >343 °C in the ELT thermal pyrolysis oils of this work, reported also by other groups,^10,69^ indicated the presence of oligomers formed from the thermal cracking of the initial polymer chains or via the recombination of free radicals. In the absence of a catalyst, the formation of aromatics has been proposed to proceed via the Diels–Alder cycloaddition of olefins and dienes to form cycloalkenes, which in turn may form BTX, MAH, and PAH species via free-radical-induced alkylation and dehydrogenation steps, favored by high temperatures and long residence times.^68,70–73^ The increased BTX yield obtained from thermal pyrolysis in the PDU (Table 4), where the residence time was longer compared to the bench-scale reactor, was attributed to this pathway.

Under the effect of the metal-free catalysts (USY, ZSM-5), accessible acid sites on the catalyst matrix or near zeolite pore openings catalyzed oligomer cracking via hydride abstraction on Lewis acid sites and protonation on Bro̷nsted acid sites followed by β-scission of C–C bonds in the resulting carbenium ions, enriching the olefin pool.^74^ The increased naphtha and middle-distillate yields and decreased heavy oil fraction yields observed in this work were consistent with this pathway. Olefins diffusing in the internal zeolite pores underwent a series of oligomerization and cyclization reactions to form cycloaliphatics, driven by the Bro̷nsted acid sites and confinement in the narrow pore network.^75–77^ Bimolecular hydrogen transfer between cycloaliphatics and olefins catalyzed on Bro̷nsted acid sites resulted in the formation of aromatic hydrocarbons.^78,79^ Successive Bro̷nsted-acid-site-catalyzed alkylation, cyclization, and hydrogen transfer steps transformed MAHs to PAHs.^80^

With a metal present on the catalyst (Ni/USY, Co/ZSM-5), *in situ*-generated reduced metal sites (Me^0^) synergistically promoted MAH and PAH formation in concert with zeolite acid sites by catalyzing dehydrogenation reactions via β-hydride elimination.^77,81^ Me^0^ sites abstracted hydrogen from olefins to yield reactive dienes and trienes that were subsequently cyclized and aromatized on nearby zeolite acid sites. Through the same mechanism, Me^0^ sites contributed to the dehydrogenation of cycloaliphatics toward aromatics and dehydrogenated alkyl-aromatics to vinyl-aromatics that served as precursors for further aromatic growth. β-Hydride elimination is characterized by H_2_ formation as a side-product, and the increased hydrogen gas yields observed primarily with Ni/USY confirmed this pathway. Increased PAH yields obtained with Ni/USY provided further confirmation. The high dehydrogenating activity of Ni/USY can be explained by the reducibility of its Ni species that initiated at temperatures below 500 °C (Figure 5) leading to the *in situ* formation of active Ni^0^ sites.

The catalytic cracking efficiency and aromatization pathways described above were strongly influenced by steric-hindrance effects. These effects can be elucidated by comparing the pore diameters of ZSM-5 (5.6 Å) and Y (7.4 Å) zeolites^61^ with the kinetic diameters of molecules representative of ELT-derived intermediates and products. Linear alkanes exhibit a kinetic diameter of 4.3 Å,^82^ while isobutane has a kinetic diameter of 5.3 Å.^83^ The kinetic diameters of rubber monomers, such as 1,3-butadiene and isoprene, would fall within this range and thus could readily diffuse into the internal pore networks of either zeolite type. Similarly, linear oligomers would have comparable kinetic diameters and could be cracked on the acid sites in the pores of either zeolite type. However, because of their longer carbon chain length, acid-site accessibility would depend on favorable alignment with the pore openings, leading to reduced reactivity in the smaller pore openings of ZSM-5. Similarly, the kinetic diameter of cyclohexane and methylcyclohexane is 6.0 Å,^82,84^ which slightly exceeds the pore diameter of ZSM-5 but is well below that of Y zeolite. As such, diffusion of cycloalkenes in ZSM-5 would be more restricted compared to that in Y. The lower heavy-oil-fraction conversion observed with ZSM-5 (Table 3, Figure 8) can be attributed to the above effects. With regard to the catalytic conversion products, the kinetic diameter of benzene, toluene, and *p*-xylene is 5.85 Å,^85^ marginally larger than ZSM-5′s pore diameter. As such, the aromatic-ring growth within the ZSM-5 pore network was limited to similarly sized molecules, resulting in the shape-selective formation of small MAH species, evidenced by the high BTX selectivity (Table 3, Figure 9B). In contrast, the larger pores of Y zeolite readily accommodated the growth and diffusion of bulkier PAH species like naphthalene (6.18 Å),^86^ phenanthrene (6.96 Å),^86^ anthracene (6.96 Å),^86^ and pyrene (7.24 Å).^86^

### 3.7 Catalyst Stability

As expected, catalysts recovered from bench-scale tests exhibited coke deposits. Coke accumulation blocks the catalyst’s pores and reduces its activity over time but can be reversed by controlled oxidation/combustion to remove organic deposits and recover catalyst activity, a standard practice in fluid catalytic cracking. Similarly, continuous operation and stable product quality in an ELT catalytic pyrolysis plant would be accomplished efficiently by the use of a circulating fluidized-bed catalytic reactor that would enable continuous coke combustion and supply of coke-free catalyst to the reaction. Alternatively, setups offering reduced complexity could employ multiple parallel fixed-bed or nontransported fluidized-bed catalytic reactors operating in alternating reaction–combustion cycles, whose frequency would be dictated by the stability of the catalyst as a function of the time-on-stream.

Beyond coke accumulation, hydrothermal effects or poisoning may irreversibly deactivate the catalyst. The results from the PDU tests presented above were obtained from triplicate tests over the same catalyst bed. Each test was followed by a combustion stage to burn off and quantify the solid products, which also regenerated the catalyst by removing coke deposits. As such, for each test point, a sequence of reaction (1)–combustion (1)–reaction (2)–combustion (2)–reaction (3)–combustion (3) steps was carried out. At the end of the final combustion step, the catalyst was recovered. A comparison of the XRD diffractograms of the catalysts before use and after three reaction–combustion cycles showed no evidence of zeolite-framework collapse (Figure S1). In combination with the good reproducibility of the triplicate tests, this indicated that the catalysts were stable, with no apparent rapid irreversible deactivation. Nonetheless, catalyst stability is crucial for process economics and must be evaluated over substantially longer operation times. Such an evaluation is critical to assess the commercialization potential and warrants a dedicated in-depth investigation that extends beyond the scope of this work.

### 3.8 Product Applications

The catalytic pyrolysis of ELTs using low-metal or Ni-poisoned equilibrium FCC catalysts in this work yielded highly aromatic oils containing up to 87 wt % aromatic hydrocarbons. These oils could be fractionated by distillation into light and heavy fractions, as reported for thermal ELT pyrolysis oils.^13^ Owing to the high aromaticity and high PAH content, the heavier fraction of these oils is especially promising as an alternative feedstock for the furnace process that produces carbon black.^14^ Carbon black is a key tire component, and its production from ELT-derived oils enables a more circular and sustainable tire industry. Sulfur in furnace process oils can be tolerated at values higher than the levels observed in the catalytic pyrolysis oils reported here,^15^ negating the need for desulfurization for this application.

From the light oil fraction, valuable BTX hydrocarbons with diverse industrial applications can be recovered. In addition, aromatic compounds in the kerosene range could be recovered from this fraction and used as sustainable aviation fuel (SAF) additives, which are typically deficient in aromatic compounds. The inclusion of aromatics in SAFs is necessary to maintain fuel system compatibility and benefits the physical and combustion properties of the overall fuel blend.^87,88^ Desulfurization of the light oil fraction may be necessary to meet the specifications for BTX and SAF-additive streams. This could be accomplished via hydrotreatment,^89,90^ which can be expected to be significantly less demanding and more selective after removal of the heavy fraction that contains most of the desulfurization-resistant benzothiophenes, dibenzothiophenes and heavier sulfur species.

Another major product from the process was ELT char, known as recovered carbon black (rCB). rCB is adulterated carbon black of inferior quality compared to virgin carbon black,^27,39^ but ongoing research into its valorization indicates its potential as a carbon black substitute for the tire industry, further enabling sustainability and circular practices.^91^

## 4 Conclusions

Catalytic pyrolysis of ELTs with equilibrium FCC catalysts (USY, Ni/USY) and ZSM-5-based catalysts (ZSM-5, Co/ZSM-5) was carried out with the objective of producing highly aromatic pyrolysis oils. The following conclusions were drawn:

1. Bench-scale testing proved effective for rapid catalyst screening, as the results closely matched those obtained from the PDU. In both setups, the most aromatic oils were obtained with Ni/USY and reached a total aromatics content of 87 wt %. Differences in vapor residence time between the two setups led to more pronounced thermal cracking in the PDU, resulting in slightly lower oil yields and increased gas production. This highlights the importance of controlling the vapor residence time to minimize secondary cracking and maximize the yields of target products.
2. The USY catalysts demonstrated the highest activity for the conversion of heavy compounds in ELT pyrolysis vapors, achieving the highest yields of BTX (up to 10.6 wt %), MAH (up to 24.2 wt %), PAH (up to 17.8 wt %), and total aromatic hydrocarbons (up to 42 wt %). The presence of Ni on Ni/USY promoted dehydrogenation reactions that increased the overall aromatic hydrocarbon yield and shifted the product selectivity toward higher MW aromatics compared to USY.
3. The ZSM-5-based catalysts showed lower overall activity but higher selectivity toward BTX and gas products. The presence of Co on Co/ZSM-5 slightly increased aromatic yields and favored higher MW aromatics through enhanced dehydrogenation reactions.
4. The most promising catalysts (USY, Ni/USY, Co/ZSM-5) were further validated in a continuous PDU, where the trends observed at bench scale were confirmed. The highest BTX yield in the PDU was achieved with USY (8.3 wt %), while Ni/USY provided the highest total aromatic hydrocarbon yield (24.8 wt % MAH, 17.1 wt % PAH, 41.9 wt % total).

In conclusion, catalyst selection was shown to significantly influence the product distribution, indicating that tailored catalyst design could further optimize yields and selectively target high-value products from ELT pyrolysis. Nonetheless, the utilization of equilibrium FCC catalysts, especially those exhibiting high Ni poisoning, offers an effective and low-cost alternative to fresh catalysts and a value-added application for this hazardous refinery waste stream. After separation of the light fraction, the high aromaticity of the pyrolysis oils from this one-step process renders them highly promising as feedstocks for carbon black production in the furnace process without the need for further upgrading. As a major tire component, ELT-derived carbon black can substantially contribute to the sustainability and circularity of the tire industry. The light fraction is also promising as a source of valuable BTX and aromatic SAF additives that can be recovered after mild desulfurization.

## Supplementary Material



## Data Availability

Data will be made available on request.

## Notes

Comparison of XRD spectra of the unused USY, Ni/USY, and Co/ZSM-5 catalysts and their used counterparts after three reaction–combustion cycles (Figure S1) (PDF)

## Abbreviations

Symbol: Meaning
BR: butadiene rubber
BTX: benzene toluene xylene
BzTh: benzothiophenes
C/F: catalyst-to-feed
CPO: catalytic pyrolysis oil
DHA: detailed hydrocarbon analysis
DiBzTh: dibenzothiophenes
ELT(s): end-of-life tire(s)
FID: flame-ionization detector
GC: gas chromatography
HPLC: high-performance liquid chromatography
IR: infrared
MAH: monoaromatic hydrocarbons
MS: mass spectrometer
MW: molecular weight
Non-Th: nonthiophenic sulfur compounds
NR: natural rubber
RT: room temperature
PAH: polyaromatic hydrocarbons
PDU: process development unit
rCB: recovered/recycled carbon black
SBR: styrene–butadiene rubber
SAF: sustainable aviation fuels
TCD: thermal conductivity detector
Th: thiophenes
TPO: thermal pyrolysis oil
TPR-H2: temperature-programmed reduction with hydrogen
XRD: X-ray diffraction
