# Supplementary material for: Chemical Recycling of End-of-Life Tires Using Catalytic Pyrolysis: Effects of Catalysts and Process Conditions toward the Production of a Highly Aromatic Pyrolysis Oil
Source: Ind Eng Chem Res. 2025 Sep 26;64(40):19342–58. doi: 10.1021/acs.iecr.5c02163 (PMC12512106; doi:10.1021/acs.iecr.5c02163)
Supplement: Supplementary file 1 [file ie5c02163_si_001.pdf]

SUPPORTING INFORMATION

Chemical Recycling of end-of-life tires using catalytic pyrolysis: effects of catalysts and process conditions towards the production of a highly aromatic pyrolysis oil

Stylianos D. Stefanidis[1,*], Eleni Pachatouridou[1], Eleni Heracleous[1,2],
Angelos A. Lappas[1], Iacovos A. Vasalos[1]

[1] *Chemical Process & Energy Resources Institute (CPERI), Centre for Research and Technology Hellas (CERTH), 6th km Harilaou-Thermi, 57001 Thessaloniki, Greece*

[2] *School of Science and Technology, International Hellenic University, 14th km Thessaloniki-Nea Moudania, 57001 Thessaloniki, Greece*

* Corresponding author: s.stefanidis@certh.gr

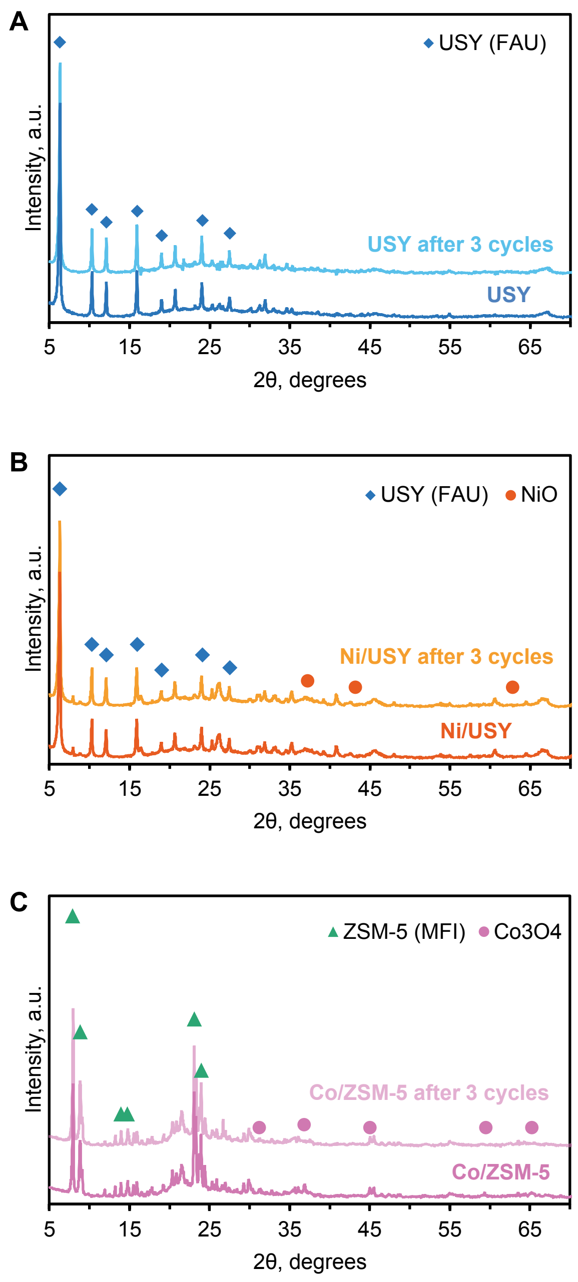

Figure S1. Comparison of XRD diffractograms of the unused USY, Ni/USY and Co/ZSM-5 catalysts with their counterparts after three cycles in the PDU.
